# ROS-Responsive Nanoplatforms for Targeted Tumor Immunomodulation: A Paradigm Shift in Precision Cancer Immunotherapy

**DOI:** 10.3390/pharmaceutics17070886

**Published:** 2025-07-05

**Authors:** Yuan-Yuan Fan, Hong Wu, Chuan Xu

**Affiliations:** 1 Chengdu University of Traditional Chinese Medicine, Chengdu 610072, China; fanyuanyuan2024@163.com; 2 Department of Oncology & Cancer Institute, Sichuan Academy of Medical Sciences, Sichuan Provincial People’s Hospital, University of Electronic Science and Technology of China, Chengdu 610072, China; wuhongzao@126.com; 3 Yu-Yue Pathology Scientific Research Center, 313 Gaoteng Avenue, Chongqing 400039, China; 4 Jinfeng Laboratory, Chongqing 400039, China

**Keywords:** reactive oxygen species, ROS-responsive drug delivery, tumor microenvironment, immunogenic cell death, antitumor therapy

## Abstract

Despite remarkable advancements in cancer immunotherapy, its clinical efficacy remains constrained in solid tumors due to the immunosuppressive tumor microenvironment (TME). Reactive oxygen species (ROS), which exhibit dual regulatory roles in the TME by regulating immunogenic cell death (ICD) and reprogramming immune cell functionality, have emerged as a pivotal therapeutic target. Nano-enabled drug delivery systems present distinct advantages for TME modulation due to their structural versatility, tumor-specific targeting precision, and spatiotemporally controlled drug release. In particular, ROS-responsive nanoplatforms demonstrate multifaceted immunomodulatory potential by synergistically restoring ICD and remodeling immunosuppressive immune cell phenotypes within the TME. These platforms further amplify the therapeutic outcomes of conventional modalities including chemotherapy, radiotherapy, and photodynamic therapy (PDT) through ROS-mediated sensitization mechanisms. This review comprehensively examines recent breakthroughs in ROS-responsive nanosystems for antitumor immunotherapy, emphasizing their mechanistic interplay with TME components and clinical translation potential. Herein, we provide a framework for developing integrated therapeutic strategies to overcome the current limitations in cancer immunotherapy.

## 1. Introduction

Composed of immunosuppressive cells—including myeloid-derived suppressor cells (MDSCs), tumor-associated macrophages (TAMs), regulatory T cells (Tregs), and cancer-associated fibroblasts (CAFs)—the tumor microenvironment (TME) establishes a hierarchical network of immune suppression [1]. These cells collaboratively obstruct CD8^+^ T cell infiltration through stromal remodeling, directly inhibit effector T cells’ cytotoxicity via cell–cell interactions, and secrete cytokines such as IL-10, TGF-β, IL-4, and IL-35 to reprogram immune cells toward tolerance [1]. Concurrently, reactive oxygen species (ROS), generated endogenously by mitochondrial dysfunction, metabolic reprogramming, or NADPH oxidase (NOX) enzymes and exogenously through hypoxia, inflammation, or therapy, exhibit concentration-dependent dual roles within this milieu [2]. While physiological ROS levels enhance antitumor immunity, their pathological accumulation disrupts the redox equilibrium, creating a self-reinforcing immunosuppressive niche [3].

At physiological levels, ROS act as signaling molecules that enhance NK cells’ cytotoxicity, T cell activation, and the antigen cross-presentation of dendritic cells (DCs) [4,5,6,7]. However, chronic oxidative stress drives tumor progression via multiple axes: ROS stabilize hypoxia-inducible factor 1-alpha (HIF-1α), allowing it to upregulate the expression of PD-L1 on tumor cells and thereby inducing T cell exhaustion [8,9,10]; mitochondrial ROS derived from hyperactive glycolysis or Lon protease overexpression reinforce Treg immunosuppression and polarize macrophages toward protumor M2 phenotypes [11,12]; and ROS play important roles in immunosuppressive stromal remodeling [13]. These opposing effects are further modulated by the ROS sources: endogenous metabolic perturbations synergize with external stimuli (e.g., hypoxia) to amplify oxidative damage, a process that accelerates metastasis and therapy resistance. Paradoxically, these effects exhibit dual roles dictated by the spatiotemporal dynamics.

Notably, the dual nature of ROS extends to therapeutic interventions, where their context-dependent effects can be harnessed or neutralized. Exogenous ROS generated using photodynamic therapy (PDT) or targeted agents induce immunogenic cell death (ICD), characterized by the release of damage-associated molecular patterns (DAMPs) such as calreticulin (CRT), adenosine triphosphate (ATP), and high-mobility group box 1 (HMGB1). These molecules recruit antigen-presenting cells to prime adaptive immunity, creating a window for synergistic immunotherapy [14]. However, the TME’s inherent oxidative stress often counteracts these benefits by sustaining immunosuppressive networks. To address this challenge, nanotechnology-based strategies are being developed to spatially regulate the ROS dynamics. Engineered nanocarriers can selectively scavenge ROS in immunosuppressive zones using catalytic materials or locally amplify the oxidative stress in tumor cells via photosensitizer delivery, all while responding to TME-specific redox gradients for controlled drug release [15,16,17].

This evolving paradigm underscores ROS as a central therapeutic target, demanding precision modulation to exploit their immunostimulatory potential while mitigating chronic oxidative suppression. Extending beyond conventional nanocarrier design, this review uniquely connects multimodal ROS-amplifying strategies—including the use of organelle-targeted generators, metal-based nanozymes, and combinatorial ROS therapies—with their direct immunomodulatory consequences across the tumor microenvironment. By integrating ROS-scavenging agents, ICD-inducing therapies, and nanotechnology-driven delivery systems, researchers are aiming to reprogram the TME’s redox landscape. Such strategies hold promise for breaking the self-perpetuating cycle of immune evasion, ultimately enhancing the effectiveness of checkpoint inhibitors and adoptive cell therapies through synergistic immunomodulation.

## 2. ROS-Mediated Immunomodulation in the TME

**Figure 1:**
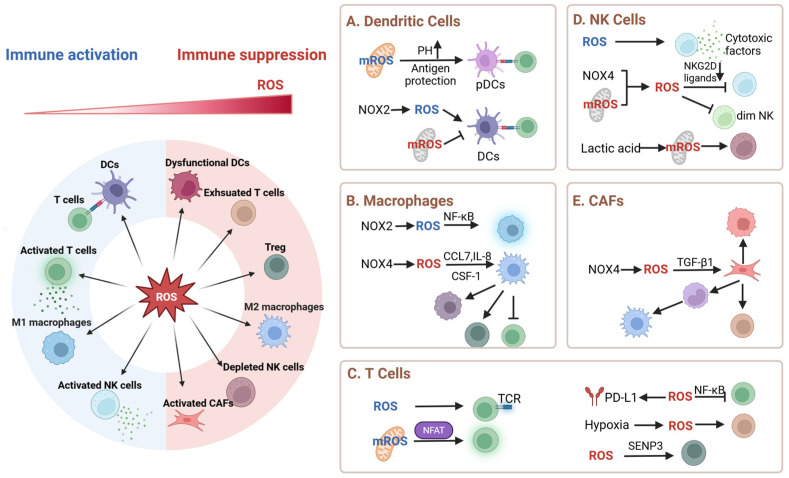
ROS-mediated immunomodulation in the TME. Created using https://BioRender.com.

ROS function as context-dependent architects of tumor immunity, dynamically sculpting both immune cells’ behavior and stromal communication networks. Their capacity to simultaneously potentiate antitumor responses and establish immunosuppressive niches creates therapeutic opportunities that are critically dependent on microenvironmental redox gradients and cellular localization (Figure 1).

### 2.1. DCs: ROS as Gatekeepers of Antigens’ Fate

ROS exert precise spatial control over DCs’ antigen processing through compartment-specific mechanisms. In phagosomal compartments, NOX2-derived ROS maintain an alkaline pH microenvironment by neutralizing the proton influx, thereby preserving antigens’ structural integrity for efficient cross-presentation to CD8^+^ T cells [6]. Conversely, within endosomal vesicles, ROS-mediated lipid peroxidation disrupts organelle membranes through the formation of oxidized phospholipid species (e.g., 4-HNE), facilitating antigens’ escape into the cytosol for subsequent MHC-I loading [18].

Plasmacytoid DCs further exploit mitochondrial ROS to optimize the cross-priming efficiency via the pH-dependent modulation of antigen degradation kinetics [19]. The temporal dynamics of ROS signaling equally determine the immunological outcomes: sustained TLR2-induced ROS production triggers lysosomal membrane permeabilization through V-ATPase disassembly, enabling antigen escape for CD8^+^ T cell priming, whereas transient ROS signals preserve the lysosomal integrity to route antigens toward MHC-II presentation pathways for CD4^+^ T cell activation [20]. Aging-associated mitochondrial ROS accumulation disrupts this delicate balance, impairing DCs’ phagocytic capacity through the oxidation of cytoskeletal regulators (e.g., cofilin inactivation) and reducing the antigen retention by 40% in aged murine models [21]. Intriguingly, acute oxidative stress can paradoxically enhance antitumor immunity through the SENP3-mediated deSUMOylation of STING, highlighting the role of ROS as context-dependent regulators of immune priming [22]. These findings collectively position ROS as molecular rheostats that calibrate DC-mediated immune priming through spatiotemporal regulation.

### 2.2. Macrophage Polarization: Redox-Driven Functional Plasticity

ROS serve as metabolic conductors orchestrating macrophages’ functional plasticity within tumor ecosystems. Pro-inflammatory M1 macrophages employ NOX2-generated superoxide anions to sustain NF-κB activation, driving the robust production of TNF-α and IL-6, which establishes antitumor inflammatory milieus [23]. In contrast, tumor-derived NOX4-produced hydrogen peroxide induces M2 polarization through HIF-1α stabilization, creating chemokine gradients (CCL7/IL-8) that recruit CCR1^+^ immunosuppressive macrophages to support tumor progression [24]. Paradoxically, while M2 macrophages promote immunosuppression through STAT3-dependent Arg1/IL-10 secretion and Treg expansion, their functional maintenance requires tightly regulated ROS levels [25]. This intrinsic redox vulnerability contrasts starkly with the antioxidant resilience of M1 populations, creating therapeutic opportunities for selective modulation. The SOD mimetic MnTE capitalizes on this dichotomy, selectively disrupting M2-mediated protumoral functions through targeted ROS scavenging while preserving the M1 cytotoxic capacity, exemplifying the precision of redox-based immunomodulation strategies [26].

### 2.3. T Cells: A Double-Edged Sword in Redox Signaling

ROS function as context-dependent rheostats of T cell immunity, balancing activation and exhaustion through spatiotemporal regulation. Physiological T cell receptor (TCR) engagement induces transient, spatially restricted ROS generation, with ROS serving as localized signaling amplifiers by oxidatively modifying key regulatory enzymes (e.g., MAPK pathway kinases/phosphatases) and redox-sensitive transcription factors (NF-κB, AP-1), thereby potentiating downstream signaling cascades and enhancing nuclear transcriptional activity to ensure sustained and robust TCR signaling fidelity [27,28]. Mitochondrial ROS (mROS) serve as metabolic amplifiers in this process, coupling enhanced oxidative phosphorylation with the nuclear translation of the nuclear factors of activated T cells (NFAT) through calcineurin activation, which drives IL-2 production and effector T cell expansion [5]. These self-limiting ROS waves ensure transient immune activation without causing oxidative damage.

In pathological contexts, chronic ROS accumulation subverts this balance through dual mechanisms: (1) sustained oxidation inactivates IκB kinase, blunting NF-κB-dependent survival signals and cytokine production [29,30]; (2) ROS-mediated post-translational modifications stabilize PD-L1 on tumor cells, creating immune-evasive niches [8,9]. Hypoxia-driven mROS further exacerbate exhaustion through pathological NFAT overactivation—a mechanism that induces a prolonged calcium influx, thereby upregulating exhaustion markers [31]. Concurrently, SENP3-BACH2 signaling enhances the Treg suppression capacity by stabilizing the expression of the transcription factor Foxp3 [11]. This redox–exhaustion axis creates therapeutic vulnerability: N-acetylcysteine (NAC) administration partially restores T cells’ functionality by rebalancing glutathione pools [32], highlighting redox homeostasis as a druggable checkpoint.

### 2.4. NK Cells: Redox Regulation of Cytotoxicity and Survival

ROS govern natural killer (NK) cells’ function through spatial–temporal redox switching. Physiological ROS gradients generated during immune synapse formation facilitate perforin/granzyme polarization through the oxidation of vesicular trafficking regulators, enabling precise cytotoxic granule delivery [7]. Paradoxically, tumor-derived ROS subvert this process through dual mechanisms: (1) the stress-induced downregulation of NKG2D ligands via HIF-1α-dependent transcriptional suppression [33]; (2) mitochondrial permeability transition pore (mPTP) activation through cyclophilin D oxidation, triggering caspase-3-mediated apoptosis [34]. This redox hijacking manifests clinically as H_2_O_2_-driven NK dysfunction—circulating CD56^dim^ subsets exhibit reduced tumor infiltration correlating with impaired ADCC activity [35]. Tumor microenvironmental stressors exacerbate this imbalance: lactate-induced acidosis in colorectal metastases provokes a change in the intracellular pH of liver-resident NK cells, causing mitochondrial ROS overproduction [36]. While activated NK cells transiently upregulate thioredoxin-1 to maintain redox homeostasis [37,38], chronic oxidative stress overwhelms these defenses, depleting liver-resident CD56^bright^ NK subsets through apoptosis [36]. These findings delineate ROS as spatial rheostats controlling NK cells’ fate, where the targeted modulation of tumor redox niches may restore the cytotoxic balance.

### 2.5. CAFs: Architects of ROS-Dependent Immunosuppression

CAFs exploit ROS to orchestrate stromal–immune collusion. NOX4-derived ROS primarily drive CAFs’ transformation into α-SMA^+^ myofibroblasts via TGF-β1/Smad2/3 phosphorylation, establishing protumorigenic extracellular matrix remodeling [39,40]. Simultaneously, ROS gradients recruit CCR2^+^ monocytes through CXCL12 upregulation, directing their differentiation into M2 macrophages and polymorphonuclear MDSCs via NOX2-dependent ERK activation [41]. These derived stromal–immune hybrids execute coordinated suppression: MDSCs deplete arginine through ARG1 overexpression, directly inhibiting CD8^+^ T cell proliferation [42], while CAF-derived monocyte exosomes deliver miR-181a to breast cancer cells, activating AKT/mTOR signaling to promote tumor progression [43]. Pharmacological NOX4 inhibition (GKT137831) disrupts this axis—reducing CAFs’ contractility and MDSC infiltration—which synergizes with anti-PD1 therapy to reverse T cell exclusion and improve tumor regression [44]. This mechanistic hierarchy positions stromal redox reprogramming as a precision strategy to dismantle architecturally complex immunosuppressive niches.

## 3. ROS and Dysregulation of ICD

ICD is a programmed cell death modality capable of activating tumor-specific adaptive immune responses in immunocompetent hosts. The current ICD inducers are classified into two categories based on their mechanisms. Type I inducers (e.g., anthracyclines) trigger apoptosis through non-endoplasmic reticulum (ER)-targeted pathways, inducing moderate ER stress and promoting the release of DAMPs such as HMGB1 and ATP to initiate DC maturation. In contrast, Type II inducers (e.g., PDT agents) directly target the ER, activating ROS-dependent ER stress via the PERK-eIF2α-ATF4 signaling axis to enhance CRT exposure and DAMP secretion, thereby significantly amplifying tumor antigens’ immunogenicity [14,45]. The core distinction between these two mechanisms lies in the ER-targeting specificity and immune activation intensity, providing a molecular basis for the precise regulation of antitumor immunity.

Traditional ICD induction approaches (chemotherapy/radiotherapy) struggle to effectively reverse the effects of an immunosuppressive TME (e.g., Treg infiltration, PD-L1 upregulation) due to systemic toxicity, poor tumor targeting, and insufficient immunogenicity [46,47]. ROS-responsive nanoplatforms address these challenges using a three-pronged synergistic strategy: ROS-sensitive materials (e.g., thioketal or arylboronic esters) enable spatiotemporally controlled drug release, increasing the intratumoral accumulation of agents like camptothecin and doxorubicin [48,49]; nanoscale encapsulation preserves DAMPs’ structural integrity, elevating DCs’ maturation rates compared to conventional methods [50]; and the co-delivery of STING agonists (e.g., cGAMP) and immune checkpoint inhibitors creates self-adjuvant systems that synergistically enhance CD8^+^ T cell infiltration and suppress MDSC activity [51,52,53]. This multi-layered engineering design achieves the systemic remodeling of the immunosuppressive microenvironment, establishing a new paradigm for next-generation combination immunotherapies.

## 4. Nanoplatform Design Strategies for ROS Amplification to Enhance ICD

**Figure 2:**
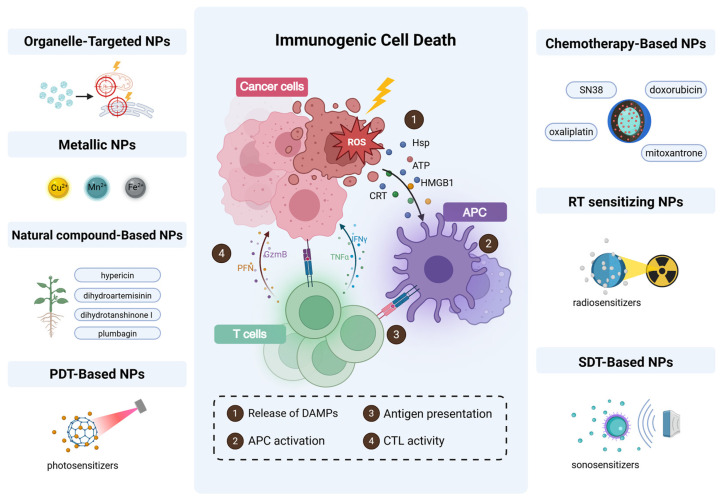
Nanoplatform design strategies for ROS amplification to enhance ICD. Created using https://BioRender.com.

Innovative nanoplatform designs synergistically amplify ROS to potentiate ICD. The strategies used span organelle-targeted delivery (mitochondria/ER), the use of metal/compound-based nanomaterials, and multimodal therapies (PDT, chemotherapy, radiotherapy, sonodynamic therapy), collectively enhancing ROS generation and antitumor immune activation. (Figure 2).

### 4.1. Organelle-Targeted ROS Delivery: Mitochondrial and ER-Centric Strategies

Mitochondria and the ER function as the central regulators of ICD, as their targeted modulation orchestrates the secretion of DAMPs and tumor-associated antigens (TAAs), thereby bridging cellular stress and systemic antitumor immunity. Mitochondria regulate ROS generation and apoptosis, while ER stress amplifies oxidative protein folding, making the mitochondria and ER ideal targets for precision ROS delivery to enhance the ICD efficacy. Organelle-directed nanoplatforms circumvent barriers to the spatial diffusion of ROS by confining oxidative bursts to defined subcellular compartments, enabling spatially confined oxidative damage and thus amplifying immune activation through precisely calibrated DAMP release.

#### 4.1.1. Mitochondria-Targeting ROS Nanogenerators

**Figure 3:**
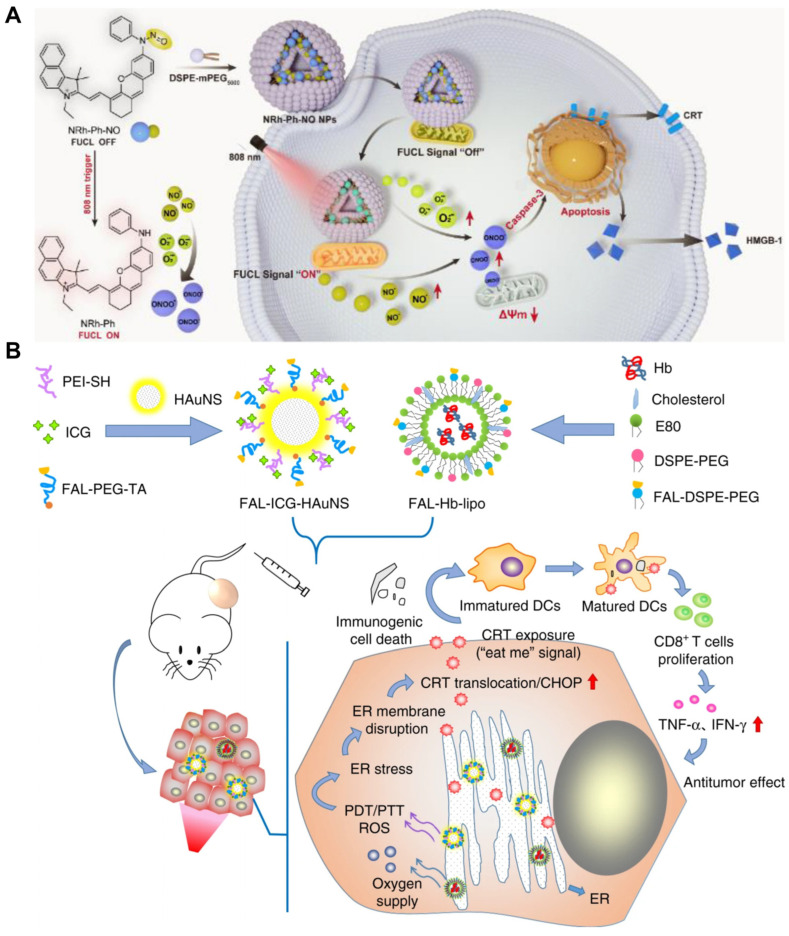
(**A**) A scheme illustrating the mechanism of mitochondria-targeted NRh-Ph-NO NPs used for in vivo NIR-light-initiated gas-mediated cancer therapy. Adapted with permission from [54]. Copyright © 2024, the authors. Published by Elsevier B.V. on behalf of the Chinese Pharmaceutical Association and Institute of Materia Medica, Chinese Academy of Medical Sciences. (**B**) ER-targeting PDT-PTT enhances antitumor immunotherapy by promoting ROS-induced ER stress and ICD activation. Adapted with permission from [56]. Copyright © 2019, the authors.

Mitochondria-targeting systems capitalize on these organelles’ heightened vulnerability to oxidative perturbation. Yu et al. demonstrated this principle using near-infrared (808 nm)-activated nanogenerators (NRh-Ph-NO NPs) that achieved the spatiotemporally controlled co-release of nitric oxide (NO) and superoxide anions (O_2_^•−^) within mitochondrial matrices [54] (Figure 3A). Phototriggered peroxynitrite (ONOO^−^) formation induced catastrophic membrane depolarization and activated executioner caspase-3, synergizing with bystander ROS to potentiate ICD in vivo. In a parallel approach, Hu et al. engineered mitochondria-homing polymer micelles (CTC NPs) co-delivering chemotherapeutic agents and photosensitizers [55]. This “all-in-one” platform enabled multistage ROS amplification via a domino effect, inducing CRT exposure, HMGB1/ATP release, and potent ICD; this cascade recruited 2.09-fold more CD3^+^CD8^+^ T cells and 1.59-fold more CD3^+^CD4^+^ T cells versus controls in tumor tissue, as evidenced by transcriptomic profiling and tumor growth suppression in murine models. These paradigm-shifting strategies exemplify the use of precision mitochondria targeting as a gateway to amplifying oxidative–immunogenic crosstalk.

#### 4.1.2. ER-Targeted ROS Amplification Systems

ER-targeted nanoplatforms leverage ER stress to enhance the ICD immunogenicity.

Liu et al. engineered pardaxin peptide-modified liposomes (Par-ICG-Lipos) for ER-localized PDT where near-infrared irradiation triggered spatially confined ROS generation within ER lumens, inducing the coordinated release of DAMPs (CRT/HMGB1) and tumor-associated antigens [57]. When combined with adoptive DCs, this ER-targeting vaccine used in PDT-based tumor treatment elicited systemic antitumor immunity, suppressing the growth of both in situ and distal tumors while establishing durable protective immunity in rechallenge models.

Deng et al. advanced the ER specificity using reduction-sensitive NPs (Ds-sP/TCPP-T(ER) NPs) featuring ER-anchored photosensitizers and glutathione-triggered payload release. Near-infrared-induced ROS amplification potentiated ICD markers, eradicating primary/distant tumors via abscopal effects while elevating the number of intratumoral IFN-γ^+^CD8^+^ T cells 2- to 3-fold versus that in controls, confirming potent antitumor T cell responses [58]. Li et al. further synergized ER-targeted PDT with photothermal therapy (PTT) using FAL-ICG-HAuNS/heme-liposomes, which reversed hypoxia via oxygen self-supply while inducing PERK-mediated CRT exposure, ultimately enhancing CD8^+^ T cell infiltration and IFN-γ secretion [56] (Figure 3B). In addition, ER-targeting PDT/PTT reduced the intratumoral Tregs to ~10%, which was not achieved in non-targeting controls, demonstrating the reversal of immunosuppressive microenvironments.

These ER-centric platforms demonstrate spatiotemporal precision in ROS generation, achieving three critical effects: maximized DAMP/antigen co-release through subcellularly confined oxidative bursts, minimized off-target toxicity via targeting, and synergistic integration with adaptive immunity through DC–T cell axis activation. Collectively, they establish organelle-specific ROS delivery as a transformative paradigm bridging nanomedicine and cancer immunotherapy.

### 4.2. Metal-Based NPs

Metal-based nanomaterials have redefined ICD paradigms through the tripartite integration of ROS amplification, TME remodeling, and multimodal therapeutic convergence [59]. These systems exploit metals’ inherent redox duality, serving as Fenton/Fenton-like catalysts for spatiotemporally confined ROS generation (via ultrasound/photoactivation) while functioning as immunomodulatory adjuvants through Cu^2+^/Mn^2+^/Fe^2+^-mediated innate immune activation [60,61]. Distinct from conventional ICD inducers, metal-based platforms circumvent biological constraints (e.g., ROS diffusion limits, immunosuppressive adenosine accumulation) by coupling metallo-specific cell death pathways (cuproptosis/ferroptosis) with cytosolic DNA sensing mechanisms, thereby orchestrating systemic antitumor immunity [62,63] (Table 1).

Luo et al. engineered a copper metal–organic framework (Cu-MOF) co-delivering Cu^2+^ and elesclomol to demonstrate copper’s dual ICD–cuproptosis synergy [61]. The acidic TME-triggered liberation of Cu^2+^–ionophore complexes induced a mitochondrial oxidative collapse via copper redox cycling, concurrently activating FDX1-mediated cuproptosis through dihydrolipoamide acetyltransferase (DLAT) oligomerization. This metallo-biological cascade elicited sequential DAMP release, driving DC maturation and cytotoxic CD8^+^ T lymphocyte infiltration, establishing copper’s capacity to synchronize metabolic cell death with the development of antigen-specific immunity. The study highlights how copper’s redox cycling capacity can synchronize cuproptosis with the development of adaptive immunity, overcoming the reliance on exogenous ionophores for cuproptosis induction. Similarly, manganese-enriched systems exploit the metal’s immunostimulatory properties. Zhou et al. designed Mn-doped zinc peroxide NPs (MONPs) that disintegrated in an acidic TME to release Mn^2+^ and generated ^•^OH via Zn^2+^-mediated Fenton reactions [64]. While ^•^OH directly induced ICD, Mn^2+^ activated the STING pathway, enhancing Type I interferon production and repolarizing immunosuppressive M2 macrophages to antitumor M1 phenotypes. This dual mechanism underscores manganese’s role in bridging oxidative stress and innate immune activation, a feat unattainable with non-metallic ROS generators.

**Figure 4:**
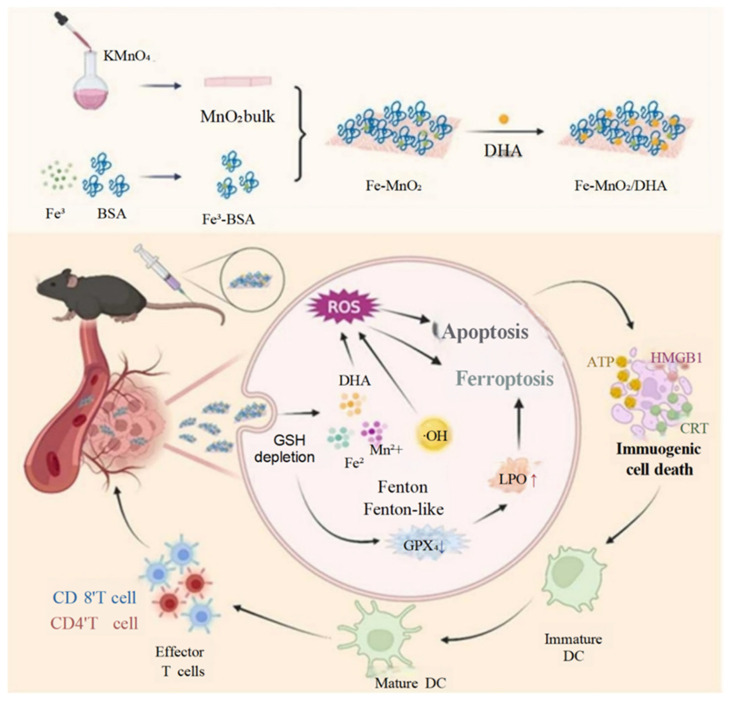
Schematic of Fe-MnO_2_/DHA NPs inducing ferroptosis, apoptosis, and ICD for synergistic hepatocellular carcinoma (HCC) therapy. Adapted with permission from [60]. Copyright © 2023, the authors. Published by Elsevier Masson SAS.

Iron-based systems further exemplify the versatility of metals in ICD induction. Huang et al.’s Fe-MnO_2_ nanosheets functionalized with dihydroartemisinin (DHA) demonstrated iron’s ferroptosis–ICD coordination. Fe^2+^/Mn^2+^-driven Fenton reactions and DHA-derived ROS cooperatively depleted glutathione and inactivated GPX4, triggering ferroptosis [60]. Concurrently, lipid peroxidation and apoptosis synergized to enhance ICD, promoting DC activation and T cell infiltration. The study illustrated how iron’s redox activity can amplify oxidative damage while linking ferroptosis to immune recognition—a critical advantage over traditional ferroptosis inducers lacking immunogenicity (Figure 4).

Beyond single-metal systems, composite platforms integrate spatial and temporal control for combinatorial therapy. Xu et al. encapsulated MnWO_x_ nanodots in gelatin microspheres (Mn-GMSs) for transarterial embolization. Post-embolization, ultrasound-triggered Mn-GMSs generated cavitation-enhanced ROS, inducing sonodynamic ICD, while Mn^2+^ release activated the cGAS/STING pathway [65]. This approach not only localized ROS generation to deep tumors but also coordinated metalloimmunotherapy with physical embolization, demonstrating the adaptability of metal-based systems to clinical procedures. Similarly, Chen et al. engineered a glutathione-responsive hydrogel (CE-Fc-Gel) incorporating ferrocene and eicosapentaenoic acid. The hydrogel’s disulfide bonds ensured the tumor-targeted release of Fe^2+^, which drove Fenton reactions and lipid peroxidation, while EPA metabolites further suppressed GPX4 [66]. Sustained ROS production and ferroptosis induction led to prolonged DC activation and systemic antitumor immunity, showcasing how metal–hydrogel hybrids enhance ICD persistence.

Collectively, metal-based nanoregulators excel in three dimensions: (1) precise ROS release—leveraging TME-responsive metal ion release to induce localized oxidative stress; (2) immune reprogramming—activating STING, repolarizing macrophages, and recruiting cytotoxic lymphocytes via metal-specific signaling; and (3) therapeutic adaptability—metal-based nanoregulators have modular designs compatible with embolization, sonodynamics, and hydrogel delivery. These advantages position metal nanomaterials as superior ICD inducers compared to organic agents, offering a roadmap for combining immunogenic death pathways with immune checkpoint inhibitors. Future research should prioritize long-term immune memory evaluation and the clinical translation of these multifunctional platforms.

### 4.3. Natural Compound-Based NPs

Natural compounds, including hypericin (Hy), dihydroartemisinin (DHA), and plumbagin (PLB), exhibit significant potential in ICD induction due to their biocompatibility, multi-target effects, and inherent immunomodulatory properties [67,68,69]. These agents directly generate ROS under stimuli such as light or enzymatic activity, triggering ER stress and mitochondrial dysfunction to promote damage-associated molecular pattern (DAMP) release. This process activates DCs and systemic antitumor immunity while minimizing the systemic toxicity, offering a translational advantage over these compounds’ synthetic counterparts.

Despite their promise, natural compounds face challenges such as poor solubility, rapid clearance, and non-specific biodistribution, which limit their therapeutic efficacy [70]. Nanotechnology addresses these limitations by enhancing tumor-targeted delivery, protecting compounds from degradation, and enabling controlled ROS generation [71,72,73]. Furthermore, integrating natural agents with complementary therapies—such as PDT, ferroptosis induction, or gas therapy—overcomes barriers posed by an immunosuppressive TME and amplifies ICD [74,75]. For example, combining ROS-generating compounds with TME-modulating agents (e.g., glutathione scavengers, oxygen suppliers) disrupts redox homeostasis, enhancing oxidative damage and immune activation.

Recent studies have highlighted innovative nanoplatforms leveraging natural compounds. Zhou et al. developed manganese oxide nano-rambutans (MnO_x_-Hy NR) encapsulating Hy, which depleted glutathione (GSH) via Mn^2+^ release while alleviating hypoxia to enhance ER-targeted PDT [64]. This dual-action system synergized ferroptosis and ICD, suppressing primary and metastatic tumors in triple-negative breast cancer models. Yang et al. engineered pH/GSH-responsive NPs (PMDC NPs) co-delivering DHA and carbon monoxide (CO), where CO amplified ROS-driven ferroptosis and apoptosis, inducing ICD in colorectal cancer [74]. Similarly, Han et al. designed biomimetic NPs co-encapsulating PLB and dihydrotanshinone I (DIH), utilizing ROS amplification to reverse the immunosuppressive TME in hepatocellular carcinoma, achieving prolonged survival without toxicity [69].

In summary, nanotechnology transforms natural compounds into precision tools for ICD induction by improving their bioavailability, spatiotemporal control, and combinatorial efficacy. These systems convert immunologically inert tumors into immunogenic hotspots through TME remodeling and adaptive immune activation.

### 4.4. PDT-Based NPs

PDT, clinically proven to be effective, employs photosensitizers (PSs), light exposure, and molecular oxygen to produce cytotoxic ROS, ultimately triggering tumor cells’ demise via apoptosis, necrosis, and ICD. Originally introduced for use in melanoma therapy, the scope of PDT has broadened to encompass various solid tumors. Nevertheless, traditional PSs, such as porphyrins and chlorins, encounter obstacles like limited aqueous solubility, aggregation-caused quenching (ACQ), hypoxia-dependent ROS generation, and inadequate tumor targeting, thereby impeding their widespread clinical application [76,77]. To surmount these obstacles, nanotechnology has stepped in, facilitating the development of ROS-responsive nanoplatforms. These platforms optimize PS delivery, exacerbate oxidative stress, and align with immunomodulatory pathways, thereby boosting ICD-induced antitumor reactions [16,17] (Table 2).

#### 4.4.1. Advanced Photosensitizer Design

Recent advances have focused on developing nanoscale photosensitizing architectures with enhanced light absorption, tunable energy transfer, and hypoxia tolerance. Metal–organic frameworks (MOFs) and covalent organic frameworks (COFs) represent promising platforms due to their ordered porosity, high surface area, and modular design. For instance, Chen et al. engineered a copper-based MOF nanocomplex (Cu-TCPP(Al)-Pt-FA) that integrated glutathione depletion via Cu^2+^ adsorption and oxygen generation using catalase-mimetic platinum NPs [78]. This dual-enhanced PDT strategy triggered ICD and reprogrammed immunosuppressive TAMs from M2 to M1 phenotypes. Similarly, Li et al. developed a photoactive metal–organic coordination polymer (MOCP) combining mitoxantrone (Mit), Ru-based PSs, and GPX4-targeting siRNA. Under 670 nm irradiation, the MOCP generated ROS to drive ferroptosis and ICD while blocking CD73-mediated ATP catabolism, thereby reversing immunosuppression and enhancing checkpoint inhibitors’ efficacy in immunologically “cold” tumors [79].

COFs, entirely organic porous materials, offer advantages over MOFs by eliminating metal toxicity risks while enabling precise functionalization. Zhang et al. designed three-dimensional COFs (COF-609) with lung-mimetic cross-linked pores to enhance ROS diffusion and ICD induction. Combined with anti-CD47 therapy, COF-609 elicited durable immune memory, as evaluated in a tumor rechallenge mouse model, and achieved >95% survival in breast cancer models [80]. Zhou et al. further engineered staggered Type I/II COF nanophotosensitizers, integrating sulfur-modified Type I motifs and Type II PSs to overcome hypoxia and ACQ [16]. This hybrid design enabled continuous ROS production under low oxygen tension, inducing potent ICD and synergizing with a PD-1 blockade to suppress metastatic progression.

#### 4.4.2. ROS Amplification Mechanisms

PDT’s efficacy hinges on two distinct ROS generation pathways: Type I, involving electron transfer-mediated free radicals such as superoxide (O_2_^•−^) and hydroxyl (^•^OH) radicals, and Type II, relying on energy transfer to produce singlet oxygen (^1^O_2_) [83,84]. Although Type II prevails under normoxic conditions, its sensitivity to hypoxia can restrict its therapeutic success. To overcome this limitation, innovative nanoplatforms have emerged, integrating Type I and II mechanisms.

**Figure 5:**
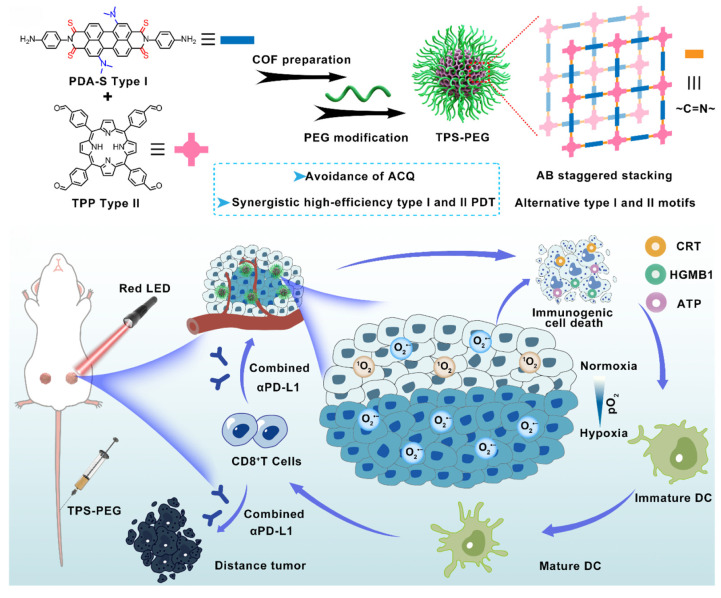
Schematic illustration of COF nanophotosensitizers with staggered Type I and II photosensitizer motifs using TPS-PEG for enhanced anticancer efficacy. Adapted with permission from [16]. Copyright © 2024, American Chemical Society.

Zhang et al., for instance, introduced a copper-embedded 3D covalent organic framework (Cu@COF-TATB) that synergistically combined photodynamic ROS generation with Fenton-like reactions [77]. This approach yielded oxidative species with dual roles, enhancing immunogenic cell death and antitumor immunity. Furthermore, when combined with aPD-1, this approach elicited systemic immune responses that inhibited abscopal tumor growth, with tumor slides exhibiting the highest levels of CD3^+^ and CD8^+^ T cells when this approach was used. Zhou et al. subsequently showed that staggered COF designs could spatially segregate Type I and II photosensitizers, facilitating concurrent O_2_^•−^ and ^1^O_2_ production even in a hypoxic TME. This potent ROS generation triggered efficient apoptosis/ICD and, when combined with ICIs, reversed tumor immunosuppression to ablate hypoxic tumors and suppress metastases through photodynamic immunotherapy [16] (Figure 5).

Moreover, plasmonic NPs, such as gold nanostars, and up-conversion nanocrystals offer additional avenues to bolster ROS generation [85,86]. These particles exploit localized surface plasmon resonance or near-infrared-to-visible photon conversion, respectively, to enhance the light penetration depth and energy utilization efficiency. These advancements are particularly vital for treating deep-seated tumors.

#### 4.4.3. Targeted Delivery and Immune Synergy

Surface functionalization employing tumor-specific ligands, such as folate or RGD peptides, or stimuli-responsive polymers sensitive to pH or redox changes augments the accumulation of photosensitizers (PSs) while reducing off-target effects. Mao and colleagues devised ROS-responsive NPs that co-deliver ectonucleotidase inhibitors (specifically, ARL67156) and PSs; these NPs selectively release their payloads within tumor sites, thereby impeding adenosine-mediated immunosuppression and amplifying PDT-induced ICD [81]. Lee et al. formulated self-assembled CPPD1 NPs that combine a cell-penetrating anti-PD-L1 peptide with a hydrophobic photosensitizer (Ce6). These NPs facilitate carrier-free tumor targeting and enhanced penetration, disrupting PD-1/PD-L1 interactions and degrading PD-L1 via lysosomal pathways [82]. When exposed to 635 nm laser irradiation, these NPs produce ROS, triggering ICD and effectively suppressing both primary and abscopal tumors by synergizing an immune checkpoint blockade with photodynamic immune activation.

### 4.5. Chemotherapy-Based Nanomaterials

Traditional chemotherapeutic agents, including SN38 and Mit, exert both direct cytotoxicity and ICD induction. This occurs through the triggering of ER stress, mitochondrial injury, and ROS generation, which leads to the release of DAMPs and subsequent activation of antitumor immunity [87,88]. These drugs can be classified into two groups: DNA-damaging agents, such as oxaliplatin derivatives, which facilitate tumor antigen release, and ROS inducers, like doxorubicin, that augment oxidative stress to boost ICD markers, including through CRT exposure and ATP secretion [89,90,91].

Despite their therapeutic potential, conventional chemotherapeutic agents face significant challenges, namely off-target toxicity, poor tumor accumulation, and immunosuppressive effects like PD-L1 upregulation [92]. Nanotechnology has stepped in as a revolutionary solution, enabling precise tumor-targeted drug delivery, controlled release over time and space, and powerful therapeutic combinations. These innovations enhance chemotherapy-induced ICD in several key ways: improved tumor targeting to reduce the systemic toxicity, locally triggered drug release in the presence of ROS for targeted oxidative damage, and the combined modulation of immune checkpoints to boost antitumor immunity. By customizing the approaches for specific drug types—for instance, targeting mitochondria with anthracyclines to increase ROS production or enhancing DNA damage using platinum agents—nanotechnology not only mitigates the inherent limitations of these drugs but also stimulates systemic immune responses, effectively bridging chemotherapy and immunotherapy [89,90,91].

**Figure 6:**
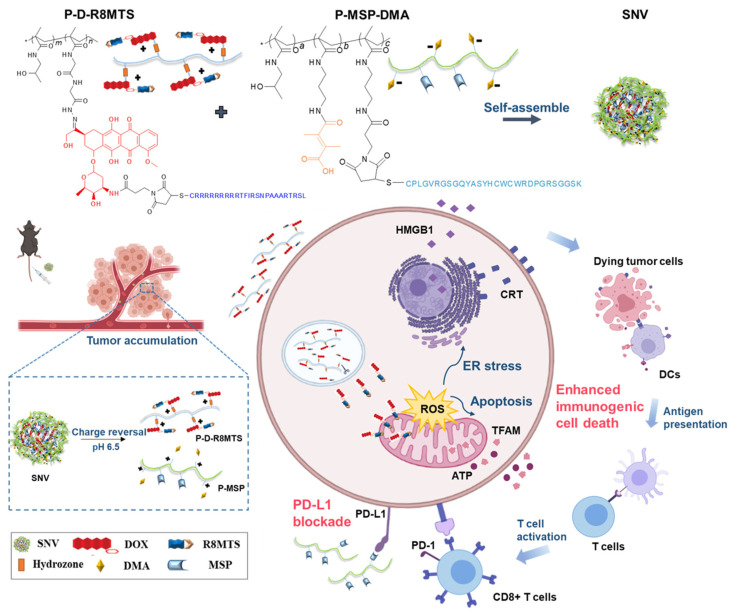
Schematic of DOX-loaded self-assembled nano vehicle (SNV) integrating mitochondrial ICD induction and PD-L1 blockade for amplified cancer immunotherapy. Adapted with permission from [92]. Copyright © 2022, Chinese Pharmaceutical Association and Institute of Materia Medica, Chinese Academy of Medical Sciences.

Doxorubicin (DOX), a well-known anthracycline, finds widespread application in ICD induction. Li et al. devised mitochondria-targeted NPs to deliver DOX, thereby boosting mitochondrial ROS levels. This elevation led to increased ER stress and mitochondrial damage markers such as TFAM. The approach potentiated CRT exposure and ATP release. However, it required the co-administration of anti-PD-L1 antibodies to counteract adaptive PD-L1 upregulation, ensuring synergistic tumor suppression in both immunogenic and non-immunogenic tumor models [92] (Figure 6). Analogously, Jeon et al. encapsulated DOX within ROS-responsive self-immolative polymers (R-SIP). Here, ROS-induced glutathione depletion disrupted redox homeostasis, augmenting ER-associated ICD and activating DCs [46]. Guo et al. employed oxaliplatin, a platinum compound, as the basis for their Nano-Folox system. This nanomedicine delivers oxaliplatin prodrugs to trigger ICD by inducing DNA damage [93]. When paired with Nano-FdUMP (loaded with the 5-Fu metabolite FdUMP), the dual nano-preparation augmented ROS generation, thereby elevating the ICD markers. This combination therapy, when used with anti-PD-L1, effectively suppressed colorectal and hepatocellular carcinomas. Moreover, it mitigated metastasis and prolonged survival in murine models. Gong et al. innovatively incorporated SN38, a topoisomerase I inhibitor, into an injectable ROS-sensitive hydrogel cross-linked with poly(vinyl alcohol) [51]. When endogenous ROS triggered hydrogel degradation, it released SN38 along with anti-PD-L1 antibodies. SN38 induced ER stress-driven ICD, while PD-L1 inhibition bolstered T cells’ responses, culminating in tumor eradication in vivo.

Mit, a derivative of anthracenedione, has been skillfully integrated into nanoplatforms to elevate ICD and antitumor immunity. In the metal–organic coordination polymer (MOCP) system developed by Li and colleagues, Mit was covalently bound to Ru-photosensitizers and complexed with Fe (II) ions, simultaneously encapsulating siGPX4 to facilitate a dual mechanism of ICD and ferroptosis [79]. Upon irradiation at 670 nm, Ru-generated ROS disrupted cellular membranes, while GPX4 silencing intensified lipid peroxidation, thus synergistically inducing both Type I and II ICD along with ferroptosis. Furthermore, the blockade of CD73 reversed adenosine-driven immunosuppression, thereby enhancing antigen presentation and inhibiting metastatic recurrence. Extending this concept, Peng and co-workers designed a tumor-targeted in situ vaccine (MBMA-RGD ISV) by linking Mit with 5-ALA via pH-responsive bonds and co-encapsulating them into MnO_2_ NPs [94]. Laser irradiation initiated a dual photodynamic–photothermal ICD response, whereas the Mn^2+^ activation of STING signaling and the alleviation of hypoxia through oxygen release reoriented macrophages toward an M1 phenotype, ultimately achieving potent antitumor immunity and suppressing metastasis.

### 4.6. Radiotherapy-Sensitizing Nanomaterials

Radiation therapy (RT) continues to be a pillar of oncology, leveraging ionizing radiation to damage DNA and produce ROS, thereby directly eliminating tumor cells [95]. Moreover, RT initiates ICD, priming the body’s antitumor immune response [96]. However, its efficacy is constrained by hypoxia-mediated resistance, off-target effects, and an immunosuppressive TME. The use of traditional radiosensitizers, such as nitroimidazoles, faces challenges due to their neurotoxicity and limited bioavailability, highlighting the need for more advanced approaches to enhance precision and safety.

**Figure 7:**
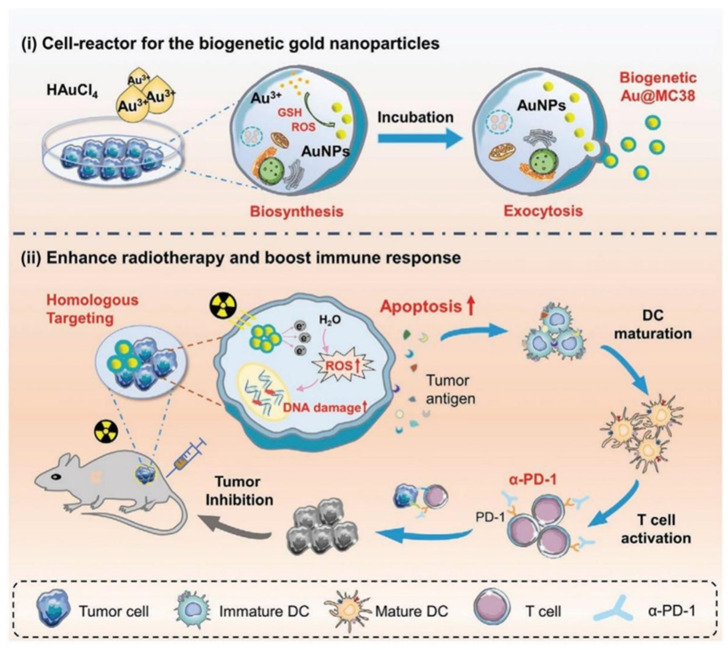
Schematic illustration of architectural design and therapeutic mechanisms of Au@MC38 in radiation-potentiated tumor regression. Adapted with permission from [97]. Copyright © 2021, Wiley-VCH GmbH.

Nanotechnology offers solutions to these issues through targeted tumor delivery, hypoxia modulation, and combined immunotherapy. Qin et al. created biogenetic gold NPs (Au@MC38) derived from tumor cells via intracellular biomineralization [97]. These NPs exploit homologous targeting to concentrate in tumors, augmenting RT-induced DNA damage and ROS generation. This, in turn, boosts ICD and activates CD8α^+^ DCs. When combined with anti-PD-1 therapy, Au@MC38 effectively suppressed primary and metastatic tumors in mouse models (Figure 7). Similarly, Huang et al. designed carrier-free gadolinium–zoledronic acid nanorods (Gd-ZA NRs) that enhance X-ray absorption for ROS generation while depleting TAMs, reprogramming the immunosuppressive TME to synergize with checkpoint inhibitors [98]. Fu et al. further developed PEGylated Cu_2_WS_4_ nanozymes (CWP) exhibiting dual Fenton-like and glutathione oxidase activities [99]. CWP potentiates RT through X-ray attenuation and nuclear penetration, while also inducing ferroptosis via the disruption of the KEAP1/NRF2/GPX4 axis. This synergizes with anti-PD-L1 therapy to hinder breast cancer progression.

Strategies focusing on hypoxia also show promising results. For instance, Gong et al. developed albumin-coated CaO_2_ NPs (CaO_2_-HSA) [100]. These NPs decompose in tumors, releasing oxygen and calcium ions. This not only alleviates hypoxia (with a sensitizing enhancement ratio of 3.47) but also triggers ICD via a calcium overload. This biosafe, metal-free system performs better than clinical radiosensitizers like sodium glycididazole, effectively halting the growth of oral cancer in situ. Such advancements highlight nanotechnology’s potential to refine RT through the integration of targeted ROS amplification, TME remodeling, and immune activation.

### 4.7. Sonodynamic Therapy (SDT)

**Figure 8:**
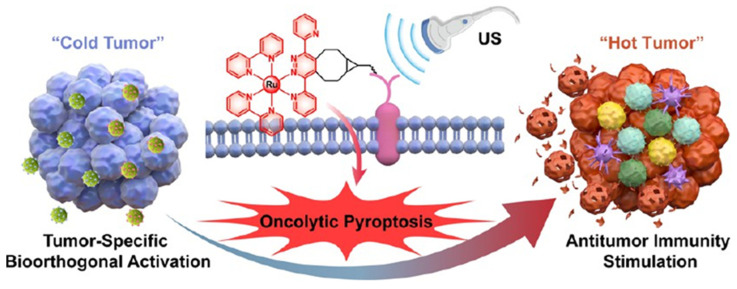
Ru (II) sonosensitizer-mediated pyroptosis/ICD activation reprograms “cold tumor” into “hot tumor”. Adapted with permission from [101]. Copyright © 2024, American Chemical Society.

SDT, which employs ultrasound (US) to activate sonosensitizers for localized ROS generation, has emerged as a promising strategy for deep-seated tumor treatment. However, traditional sonosensitizers suffer from poor tumor targeting, a limited ROS quantum yield, and the insufficient modulation of immunosuppressive TMEs. Recent advancements in nanotechnology have addressed these limitations through the rational design of ROS-responsive nanoplatforms that enhance SDT’s efficacy while robustly inducing ICD. For instance, Xu et al. developed bioorthogonal Ru (II) sonosensitizers that selectively anchor to tumor membranes via metabolic glycoengineered BCN receptors, enabling precise US-triggered ROS generation [101]. This membrane-targeted approach induced pyroptosis, leading to the release of DAMPs and the activation of systemic antitumor immunity within immunologically “cold” tumors (Figure 8). Also, Peng et al. (2025) designed dual-targeting aggregation-induced emission (AIE) polymer micelles (ABM-M) that optimized ROS production when exposed to US irradiation [102]. These micelles not only initiated ICD but also facilitated the reprogramming of immunosuppressive M2 macrophages into pro-inflammatory M1 phenotypes, thereby efficiently overcoming TME immunosuppression.

Additional advancements include oxygen-deficient nanosensitizers, such as MoO(X)-PEG, crafted by Wang and colleagues. This agent depletes glutathione and triggers cGAS-STING pathways via excessive ROS production [103]. Its synergy with anti-CTLA4 therapy bolsters DC maturation and cytotoxic T cell infiltration, thereby markedly impeding metastatic progression. Chen’s team, on the other hand, merged bimodal imaging (^19^F MRI/CEUS) with pH-sensitive liposomes (LP@PFH@HMME). This union facilitated a “nano-to-micro” shift in an acidic TME, amplifying ROS generation [104]. This approach, when combined with a PD-L1 blockade, potently curbed the recurrence of triple-negative breast cancer by enhancing ICD induction. Furthermore, Jiao and co-workers underscored the potency of oxygen-deficient ZrO_2-X_ nanoplatforms in merging PTT with SDT. In their study, ROS-induced ICD elevated the level of pro-inflammatory cytokines, such as TNF-α and IFN-γ, ultimately fostering DC activation [105].

Further advancements underscore the adaptability of nanoplatform design. Yin et al. employed Listeria-tropic microbial nanosonosensitizers (Bac@ARS) to address tumor hypoxia, thereby augmenting ROS production and ICD-mediated antitumor immunity [106]. Xu et al. encapsulated MnWO(X) nanodots within gelatin microspheres (Mn-GMSs), harnessing ultrasound-induced cavitation to elevate ROS generation and cGAS/STING activation, ultimately enhancing the efficacy of PD-L1 blockades [65]. Liu et al. crafted ultrasound-activated PROTAC prodrugs (NP(Ce6+PRO)) capable of degrading the oncoprotein BRD4 while mitigating PD-L1 upregulation, providing precise spatiotemporal control over ICD and immune checkpoint inhibition [107]. Collectively, these investigations illustrate how nanotechnology-powered SDT platforms not only amplify ROS generation but also reshape immune environments via ICD, presenting a revolutionary opportunity for cancer immunotherapy.

## 5. ROS-Responsive Nanoplatforms for Targeted Immunomodulation

ROS-responsive nanoplatforms have emerged as precision tools to dismantle immunosuppressive networks in the TME. These systems exploit the elevated ROS levels in tumors to achieve spatiotemporal control over drug release, selectively targeting immune cells while minimizing off-target effects. By integrating stimuli-sensitive linkers (e.g., thioketal, selenium, or arylboronic esters) with immune-modulating cargo, such nanocarriers enhance antigen presentation, reverse T cell exhaustion, reprogram macrophage polarization, and disrupt CAF activity [108,109,110]. Their design often incorporates dual functionality—scavenging immunosuppressive ROS while delivering therapeutics—to synergize with endogenous immune activation pathways. This section highlights recent breakthroughs in ROS-responsive nanosystems tailored to regulate DCs, T cells, macrophages, and CAFs.

### 5.1. Restoring DC Antigen Cross-Presentation

DC dysfunction, a characteristic feature of ROS-rich TMEs, can be mitigated using nanoplatforms designed to bolster antigen processing and co-stimulatory signaling. Xie et al. revealed that, surprisingly, intracellular ROS kinetics dictate antigen presentation modes, with steady, moderate ROS production elevating cross-presentation [20]. To this end, they devised a 3D scaffold vaccine incorporating the TLR2 agonist acGM alongside tumor antigens. This scaffold, utilizing an ROS-degradable polysaccharide structure, maintained TLR2 activation in DCs, inducing intracellular ROS generation through NOX2 coupling. This controlled oxidative stress facilitated antigen release from endosomes via lipid peroxidation, thereby enhancing MHC-I cross-presentation and the priming of CD8^+^ T cells in murine models of lung metastasis. Likewise, Wang et al. designed pH-responsive galactosyl–dextran–retinol (GDR) nanogels specifically targeting DCs [111]. These nanogels enhance MHC-I antigen cross-presentation by inducing lysosomal rupturing and activating the proteasome in an ROS-dependent manner. These self-adjuvanted nanogels aid DC maturation and cytosolic antigen release and elicit potent antitumor immune responses in vivo, presenting a new approach to enhance the effectiveness of cancer vaccines.

### 5.2. Reinvigorating T Cell Activation

ROS-responsive systems effectively counter T cell exhaustion by suppressing immune checkpoints and alleviating metabolic repression. Zhao and colleagues devised a DNA nanocage capable of simultaneously delivering chloroquine, an autophagy inhibitor, and CpG oligonucleotides [112]. This nanocage selectively disintegrates in high-ROS environments, thereby unleashing CpG to stimulate TLR9 on DCs, while chloroquine sustains the MHC-I expression on tumor cells. This system synergistically restores cytotoxic T cell immunity, demonstrating potent antitumor effects in preclinical pancreatic adenocarcinoma models. In a separate strategy targeting direct T cell modulation, Gao et al. introduced cationic selenium nanogels (A_1.8_Se_3_O_0.5_/siPD-L1) designed to degrade in the presence of ROS, releasing PD-L1 siRNA [108]. These nanogels concurrently elevate the lysosomal pH to hinder autophagy-induced PD-L1 upregulation, ultimately reinstating MHC-I expression and bolstering T cells’ cytotoxic activity against MC38 tumors.

### 5.3. Reprogramming Macrophage Polarization

Nanoplatforms aimed at disrupting ROS–metabolic crosstalk in macrophages effectively redirect M2-like TAMs towards an antitumor phenotype. Yang and colleagues designed Type I photosensitizers, such as TBZ, capable of producing extracellular ROS through electron transfer when exposed to near-infrared radiation [113]. These ROS trigger the activation of the NLRP3 inflammasome in TAMs, leading to a downregulation of Arg1 and CD206 and an upregulation of MHC-II and TNF-α. This process successfully converted M2 macrophages to M1 macrophages in breast cancer models. Mo et al. developed a Zr-CeO nanozyme with dual superoxide dismutase and catalase enzymatic activities, effectively scavenging ROS to suppress STAT3 phosphorylation in TAMs [114]. In preclinical renal and breast cancer models, Zr-CeO synergistically enhanced the PD-1 blockade efficacy by reducing MDSC infiltration and polarizing TAMs toward an immunostimulatory state, while also promoting intratumoral T cell recruitment and IFN-γ production.

### 5.4. Disrupting CAF-Mediated Immunosuppression

CAFstargeted nanotherapies effectively counteract the ECM remodeling and cytokine secretion driven by ROS. Alili et al. showed that cerium oxide NPs (CNPs) effectively scavenge ROS in squamous cell carcinoma. This inhibits the differentiation of CAFs into myofibroblasts and prevents the stiffening of the ECM mediated by TGF-β1, which creates a physical barrier that impedes T cell penetration [15]. In parallel, Gong et al. engineered a cancer cell microvesicle-inspired polyfluorocarbon nanosystem (M-FDH) co-delivering the radiosensitizer DiIC18(5) (DiD) and antifibrotic halofuginone (HF). By alleviating tumor hypoxia and remodeling the stromal architecture, M-FDH synergized with radiotherapy to amplify tumor cell eradication and antitumor immunity. Notably, this strategy achieved a 90% reduction in the CAF density and markedly elevated CD8^+^ T cell infiltration in 4T1 breast tumors, highlighting its dual therapeutic–immunomodulatory potential.

## 6. Discussion and Future Perspectives

The emergence of ROS-responsive nanosystems has profoundly transformed tumor immunomodulation, offering precise spatiotemporal therapeutic delivery, amplifying ICD, and reshaping immunosuppressive TMEs. These platforms harness the abnormal redox states of tumors to selectively unleash immunotherapeutics, chemotherapy agents, or photosensitizers, thereby bolstering antitumor immunity and minimizing collateral damage. Notably, organelle-specific ROS generators, like mitochondrial or ER-targeted nanosystems, exhibit remarkable ICD induction via the creation of localized oxidative stress, whereas metal-based nanomaterials employ Fenton-like reactions and immunostimulatory ions to link oxidative harm with innate immune activation. Combination strategies that merge PDT or SDT with checkpoint inhibitors have proven effective in converting immunologically “cold” tumors to “hot” ones by coupling ROS-driven ICD with immune checkpoint inhibition. The versatility of these nanosystems—ranging from natural compound carriers to cutting-edge photosensitizing frameworks—highlights their adaptability in tackling a range of immunosuppressive mechanisms, including DC dysfunction, T cell exhaustion, and M2 macrophage polarization. In sum, ROS-responsive nanosystems mark a seismic shift in cancer immunotherapy, offering multifaceted strategies to elevate therapeutic precision and immune coordination.

Despite some nanoformulations having entered clinical evaluation (Table 3), many trials have been terminated due to insufficient efficacy or unacceptable toxicity [115,116]. Significant translational barriers persist for ROS-responsive nanotherapies, primarily concerning nanomaterials’ biocompatibility and long-term biosafety. For instance, metal-based systems, potent in ROS production and immune modulation, may cause unintended cytotoxicity due to a metal ion buildup or off-target redox reactions. Organic carriers, like thioketal or selenium-containing polymers, face uncertainties regarding their metabolic clearance and potential immunogenicity. The intricate heterogeneity of the TME further complicates targeted delivery, as varying ROS levels across tumor regions can lead to uneven drug release and suboptimal immune activation. Additionally, preclinical models often fail to fully capture the complex dynamics between human immune systems and nanotherapeutics, limiting their predictive power for clinical outcomes. Ensuring scalability and reproducibility in manufacturing also poses challenges, especially for multifunctional nanosystems requiring exact stoichiometric control over components such as photosensitizers, immunoadjuvants, and targeting ligands. Moreover, while combination therapies hold promise, they also carry the risk of exacerbating systemic inflammation or autoimmune reactions, necessitating rigorous safety assessments.

Research efforts should prioritize the development of more intelligent nanosystems incorporating real-time imaging and feedback loops to dynamically adjust the ROS generation and drug release in accordance with TME changes. Breakthroughs in biomimetic coatings, like cell membrane-derived vesicles, could potentially enhance tumor targeting while allowing for the evasion of immune detection. Furthermore, harnessing the power of artificial intelligence in rational nanomaterial design may expedite the discovery of biocompatible, stimulus-responsive carriers with optimized pharmacokinetic profiles. Clinically, longitudinal studies are crucial to evaluate the long-term toxicity and immune memory formation, while patient stratification based on redox biomarkers may pave the way for personalized nanotherapy. Collaborative endeavors between material scientists, immunologists, and clinicians are essential to bridge the divide between innovative nanosystems and transformative cancer treatments, ultimately unlocking the full potential of ROS-responsive immunomodulation in the fight against cancer.

## Figures and Tables

**Table 1:** Metal-based NPs.

Drugs/NPs	Cancer Model	Antitumor Mechanisms	Immunity Activation In Vivo	Ref.
Fe-MnO_2_/DHA	HCC	ICD, ferroptosis, apoptosis	~3.36-fold increase in CD8^+^ T cell infiltration; promotes macrophage polarization	[60]
ES-Cu-MOF	Fibrosarcoma	ICD, cuproptosis	~1-fold increase in CD8^+^ T cell infiltration; systemic antitumor immunity	[61]
Cel-Cu	Breast cancer	ICD, cuproptosis	~0.5-fold increase in CD8^+^ T cell infiltration; promotes the polarization of TAMs	[62]
MONPs	Breast cancer	ICD, activate STING pathway	~1.83-fold increase in CD8^+^ T cell infiltration in spleen; reduces Tregs; polarizes M2 macrophages to M1 type	[64]
Mn-GMSs	Liver cancer	ICD; activate cGAS-STING pathway	~1.21-fold increase in CD8^+^ T cell infiltration	[65]
CE-Fc-Gel	Breast cancer	ICD, ferroptosis, apoptosis	Promotes the maturation of DCs; increases the infiltration of CD8^+^ T cells	[66]

**Table 2:** PDT-based NPs.

Drugs/NPs	Cancer Model	Antitumor Mechanisms	Immunity Activation In Vivo	Ref.
Cu-TCPP(Al)-Pt-FA	Lung cancer	ICD	~5-fold increase in CD8^+^ T cell infiltration; polarizes M2 macrophages to the M1 type	[78]
MOCPs	Breast cancer	ICD, ferroptosis	Increase in cytotoxic T cell infiltration	[79]
COF-609	Breast cancer	ICD	Increase in the ratio of T cells	[80]
3D Cu@COF-TATB	Breast cancer	ICD	Increase in cytotoxic T cell infiltration	[77]
TPS-PEG	Breast cancer	ICD, apoptosis	~0.83-fold increase in CD8^+^ T cell infiltration	[16]
NP700-ARL	Oral cancer, colon cancer, breast cancer	ICD	~2.59/2.7-fold increase in IFN-γ–CD8^+^ T cell infiltration	[81]
CPPD1	Colon cancer	ICD	Increase in tumor-responsive T cells; blocks PD-1/PD-L1 interactions	[82]

**Table 3:** Clinical trials of ROS-responsive nanoplatforms for cancer treatment.

Drugs/Nps	Cancer Type	Combination Therapy	NCT	Phase	Ref.
NBTXR3	HNSCC	RT/cetuximab	NCT04892173	Phase III	[117]
NBTXR3	Soft-tissue sarcoma	RT	NCT02379845	Phase II/III	[118]
Anti-EGFR-ILs-dox	TNBC	-	NCT02833766	Phase II	[116]
Anti-EGFR-ILs-dox	Glioblastoma	-	NCT03603379	Phase I	[119]
NLG207	Prostate cancer	Enzalutamide	NCT03531827	Phase II	[115]
NLG207	NSCLC	-	NCT01380769	Phase II	-
